# Use of Unpalatable Forages by Ruminants: The Influence of Experience with the Biophysical and Social Environment

**DOI:** 10.3390/ani8040056

**Published:** 2018-04-14

**Authors:** Roberto A. Distel, Juan J. Villalba

**Affiliations:** 1 Centro de Recursos Naturales Renovables de la Zona Semiárida (CERZOS), Departamento de Agronomía, Universidad Nacional del Sur (UNS)-CONICET, La carrindanga km 7, Bahía Blanca 8000, Argentina; 2 Department of Wildland Resources, Quinney College of Natural Resources, Utah State University, Logan, UT 84322-5230, USA; juan.villalba@usu.edu

**Keywords:** unpalatable forages, ruminants, environmental experience, early experience, diet selection, habitat selection

## Abstract

**Simple Summary:**

Unpalatable forages, due to either low nutrient content or the presence of toxic compounds, are widespread and represent a challenge for ruminant nutrition, health, and welfare. If we find ways to encourage consumption of unpalatable forages, they could provide at least part of the nutrient requirements of ruminants. Our objective was to synthesize the role of diverse environmental experiences on the use of unpalatable forages by ruminants. Experimental evidence shows that experience can alter both morpho-physiological and psychological (learning) mechanisms to better cope with unpalatable forages, particularly early in life when body functions are more amenable to change. Furthermore, experiential learning provides flexibility in diet selection, which is critical in changing foraging environments. By understanding and applying behavioural principles, it is possible to better devise management plans that optimize the nutrition, health, and welfare of herbivores grazing unpalatable forages throughout their life. In addition, a more uniform use of resources can be achieved from the landscape level down to the individual plant, with consequent benefits to ecosystem integrity and stability.

**Abstract:**

Unpalatable forage resources (low nutrient density, potentially toxic metabolites) are widespread and represent a challenge for ruminant nutrition, health, and welfare. Our objective was to synthesize the role of biophysical and social experience on the use of unpalatable forages by ruminants, and highlight derived behavioural solutions for the well-being of soils, plants, and animals. Environmental experiences early in life modulate gene expression and promote learning, which alters morpho-physiological and psychological mechanisms that modify behavioural responses and change food and habitat selection. In this process, ruminants can become better adapted to the habitat where they are reared. Moreover, experiential learning provides flexibility in diet selection, which is critical for changing foraging environments. Learned associations between unpalatable and palatable foods, if ingested in appropriate amounts, sequence, and close temporal association, induce the development of preference for the former type of food. In this way, a more uniform use of resources can be achieved from the landscape level down to the individual plant, with the associated benefits to ecosystem integrity and stability. Ruminants can also learn the medicinal benefits of ingesting foods with toxins (e.g., condensed tannins and saponins with antiparasitic properties). This knowledge on behavioural processes can be translated into behavioural applications that provide low-cost solutions to many challenges that producers face in managing sustainable livestock production systems.

## 1. Introduction

Ruminants are frequently faced with grazing unpalatable forages, which represents a challenge for them due to either low-quality (low nutrient density) or the presence of toxic compounds. Animals deal with this problem through evolved morpho-physiological mechanisms [1,2] and by means of experience. Experience can alter both the morpho-physiological and psychological (learning) mechanisms to better cope with unpalatable forages [3,4,5,6,7].

Experience allows for flexibility in diet selection, which is crucial in changing foraging environments. Ruminants are born with an inherent ability to learn about food [8], and they learn by individual and social experience. Individual learning theory argues that animals are able to associate food sensorial characteristics (taste, odour, aspect) with its post-ingestive consequences, and that this associative learning process is used to modulate preference or aversion for foods, which determines diet selection. The underlying mechanism of learning about the post-ingestive effects of specific foods encompasses the interaction between affective and cognitive processes. Affective processes involve the integration of a food’s taste with post-ingestive feedback from cells and organs in response to the levels of ingested chemicals. This integration occurs automatically [9] and causes changes in the liking of food items that depend on whether the effect on the internal environment is aversive or positive [10]. The net result is incentive modification. On the other hand, the cognitive system integrates the odour and sight of food with its taste. Animals use their sense of smell and sight to differentiate between different types of food, and to select or avoid food whose effect on their internal environment is either positive or aversive. The net result is behaviour modification. Together, affective and cognitive processes enable animals to maintain fluidity in their dietary decisions, given the ongoing changes in the internal and external environments [11].

Animals also learn about food indirectly through in utero and neonate experiences and by observing conspecifics [12]. The development of food preferences begins at the foetal/pre-ruminant stages when the young animal experiences the flavours of food consumed by its mother [13,14,15]. Later on, when the animal starts to ingest solid food, observation of conspecifics represents a way of learning food preferences [16] and of discriminating between food differing in post-ingestive consequences [17,18]. At this stage in life, the mother stands in as the best social model [19].

Food experience, particularly early in life (see below), can change morpho-physiological mechanisms and hence the post-ingestive consequences, modifying the incentive value for food already experienced and thus its intake value. Young ruminants exposed to low-quality food (high fibre content, low nitrogen content) or toxin-containing food (e.g., phenolic compounds) early in life can develop morphological (e.g., larger reticulum-rumen size) and/or physiological (e.g., better nitrogen economy, greater detoxification capability) mechanisms that enhance the acceptance of and preference for these types of food [3,4,5]. On the other hand, the context in which animals experience unpalatable forages can result in learned associations that enhance consumption of and preference for them [20,21,22]. For instance, sheep can develop a preference for either low-quality [6,7] or toxin-containing [23] food when this food precedes a highly nutritious and preferred food in a sequence familiar to the animal.

Our objective was to synthesize the role of diverse environmental experiences on the use of low-quality feed and toxin-containing feed by ruminants, with the aim of stimulating novel approaches for improving animal production and land integrity in systems that include unpalatable forage resources.

## 2. The Importance of Experience in Ruminant Animals

**Figure 1:**
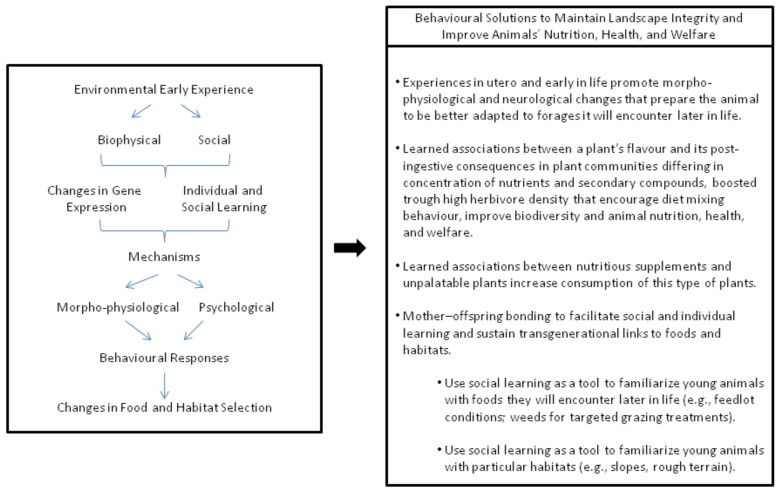
Conceptual framework on the influence of experience with biophysical and social environments in the development of food and habitat preferences by ruminants, and derived behavioural solutions for the well-being of animals, plants, and soils.

Environmental experiences, starting in utero and continuing early in life, cause a suite of neurological [24,25,26], morphological [27], and physiological [28] changes that affect behaviour in ways that enable animals to thrive in a world of change (Figure 1) [11]. By interacting with the genome during growth and development, social and biophysical environments influence gene expression and behavioural responses, as the emerging field of epigenetics has highlighted. Over generations, these interactions create animals that are locally adapted to the landscapes they inhabit [29]. This dynamic dialogue between the environment and the genome, crystallized in the animal’s physiology and behaviour, implies that while the body influences the structure and function of experience (genetics), it is just as true that experience influences the structure and function of the body (epigenetics) [11]. The epigenome entails those heritable chemical changes to the genome (e.g., DNA methylation or histone modification) that modify gene expression without changing the DNA sequence.

Learning from mother begins early in the developmental process and can have lifelong effects when it comes to forage preferences. Recent research suggests that mothers have a powerful and lasting influence on their offspring even before birth. Pregnancy is not an incubation period but a staging period for well-being and disease later in life [30,31]. A better understanding of the developmental processes that take place in utero and of the effects they impinge on herbivores later in life may aid in the development of novel management plans that use grazing animals to achieve their full potential as “landscape architects.”

The concept of foetal programming was first hypothesized for humans using epidemiological data, which suggested that the uterine environment in undernourished mothers altered the long-term development, growth, and susceptibility to disease in their offspring (i.e., the ‘Barker hypothesis’ [32,33]). The role of early environmental events, acting as epigenetic factors, on the “programming” of behavioural responses in mammals was then unveiled for stress responses in rats [34,35] and subsequently for other species including humans [36,37]. Since then, it has been shown that management of maternal nutrition influences foetal organ development, muscle development, postnatal calf performance, carcass characteristics, and reproduction in livestock [38]. Early-life programming may then represent a management tool that offers a faster approach than genetic selection for responding to environmental contingencies in the short-term and potentially modify dietary preferences in ruminants.

The aforementioned findings highlight the importance of mothers as a transgenerational link to the food their offspring are likely to eat and the habitats they are likely to inhabit [10,39]. With few exceptions [40,41,42], the aforementioned studies conservatively estimate the degree to which experience early in life affects the performance of adults due to testing having occurred when animals were young and still learning about food, rather than as adults years later [43]. These processes, which enable animals to adapt to diets and habitats locally, imply that what constitutes a “high quality diet or habitat” will differ for herbivores reared in different environments and at different points in space and time. Thus, the “absolute fitness value” or “nutritional quality” for a certain food may change as a function of an animal’s early experiences with that food [22].

Social interactions and locally adapted cultures are an essential part of the collective memory of a species that individuals learn from their ancestors through their mothers. That knowledge, locally inflected through environmental experiences, adds uniquely to the biodiversity of landscapes and it has clear implications for conservation [44,45], animal nutrition [46], health [47], and welfare [48,49]. When we sever transgenerational links to landscapes by moving animals to unfamiliar habitats, both wild and domestic herbivores suffer more from predation, malnutrition, over-ingestion of poisonous plants, and poor reproductive performance [50].

## 3. Positive Experience and Use of Low-Quality Feed

The most striking results of the influence of experience on the use of low-quality feed by ruminants come from exposure to this type of food early in life, when neurological, physiological, and morphological processes are amenable to change and can be altered so that animals can better forage in the habitat in which they are reared [51]. Such adaptation in mammals can be achieved through epigenetic mechanisms occurring in utero and early in life [52,53]. Experiences early in life with their mothers consuming low-quality forages can have a long-life influence on acceptance of, and preference for, this type of food. For instance, lambs that experienced low quality roughage (cured weeping lovegrass) with their mothers until weaning ingested 20% more mature sorghum hay (low-quality forage) immediately after exposure than inexperienced animals and, when given a choice between alfalfa hay (high-quality forage) and sorghum hay, the experienced animals ate 2.5 times more sorghum than the inexperienced lambs [4]. The differences between experienced and inexperienced animals in acceptance of, and preference for, sorghum hay persisted up to nine months after completion of the initial exposure [5]. Experienced lambs showed higher apparent digestibility of sorghum hay (4.5% higher) and a better nitrogen retention than inexperienced lambs, which helped to explain the observed feeding behaviour. Similarly, suckling female calves exposed to ammoniated wheat straw for 66 days showed a better productive performance as cows when re-exposed to ammoniated wheat straw from 5 to 8 years later in life compared with naïve cows [42]. Calves whose dams ate ammoniated wheat straw showed a higher digestibility and intake of this forage than calves whose dams did not eat any ammoniated wheat straw [54]. Previous studies showed that ruminants can learn preferences for food experienced early in life, and that such behaviour persists over time [55].

Likewise, experience early in life with the unpalatable weed medusahead (*Taeniatherum caput-medusae* ssp. *asperum*) can improve animals’ performances when fed on this low-quality forage (high silica concentration) as adults. Yearling sheep that experienced medusahead early in life with their mothers showed a more even intake of medusahead across days and a greater gain-to-feed ratio than the control yearlings, which was attributed to possible greater number of rumination events and greater in vivo digestibility values in experienced animals [56]. Moreover, the relative extent of use of an unpalatable weed (medusahead) by mothers (low, medium, or high) was reflected in the use of medusahead by their offspring [57]. Thus, it may be possible to capitalize on this individual variation for targeted grazing treatments by selecting females—either through genetic markers or observation—that show a high propensity to consume medusahead, as most likely they will have offspring with similar dietary habits. Alternatively, a diet composed of an invasive weed may influence the gut microbiome, which in turn may contribute to promoting changes in the host’s phenotype (see below, “Experience and the gut microbiome”). Taking this variable into account could potentially enable us to identify shifts in microbial populations that promote a more efficient utilization of unpalatable species, selecting animals that manifest such “positive” shifts, as well as to manipulate and design rumen microbial communities in the future.

A clear demonstration of learned associations underlying experience with low-quality food in a nutritionally rich context comes from controlled trials with sheep, with results consistent with the idea that acceptance of and preference for a particular food is not absolute but relative to the nutritional context in which the food is consumed. For instance, intragastric infusion of nutrients can condition a preference towards a low-quality feed (e.g., wheat straw) in sheep [58,59]. Sheep showed enhanced intake of, and developed a preference for, oat straw when its consumption was immediately followed by consumption of nutritious food (e.g., soybean meal or corn) [6,7]. In the latter studies, the design of the experiments suggested that a learned association between oat straw and the post-ingestive feedback from the nutritious supplements was the fundamental reason for the sheep’s intake induction and increased preference for oat straw. However, the persistence of intake induction through conditioning was short-lived, since when the provision of the nutritious food was discontinued the intake of oat hay converged with values comparable to those observed in the control animals. Moreover, no difference has been observed in the intake of low-quality food between conditioned and unconditioned (i.e., controls) animals in associating the ingestion of a low-quality feed with the subsequent presentation of a nutritious alternative [60,61]; however, this does not necessarily mean that learning has not taken place. The expression of learning is context-dependent, as differences in foraging effort (e.g., constraints in food availability, differences in handling and searching times) may unveil foraging patterns that are not observed when high- and low-quality foods are highly available and thus offer similar foraging efforts. For instance, when conditioned sheep were faced with a choice between a target low-quality food and a high-quality alternative under conditions when foraging efforts to gather those foods were similar, they displayed an almost absolute preference for the high-quality food, similar to values observed for the control animals. However, when access to the high-quality food was restricted in a foraging arena, intake and selection of the low-quality food was much greater in the conditioned animals that previously experienced the ingestion of the low-quality food in association with a high-quality food than the control sheep, which had not experienced such an association [60,61].

These results of the influence of experience with low-quality food in a ruminant’s early life reveal that both morpho-physiological and psychological mechanisms underlie the observed changes in feeding behaviour.

## 4. Positive Experience and Use of Toxin-Containing Feed

Experience also influences the use of toxin-containing plants. Distel and Provenza [3] found that goat kids that browsed on blackbrush (*Coleogyne ramosissima*, low in nutrient density and high in condensed tannins) with their mothers ingested nearly twice as much blackbrush as inexperienced goat kids fed with alfalfa pellets with their mothers, immediately after weaning. Nine months after exposure, experienced goats still ingested 30% more blackbrush than inexperienced goats, and they showed an increased preference for blackbrush when offered in a choice with alfalfa pellets. Experienced goats were better able to detoxify phenols (excreted 63% more uronic acid than inexperienced goats) and had increased their reticulum-rumen capacity one month after exposure to blackbrush. Likewise, lambs from ewes that grazed saltbush (*Atriplex nummularia*, with approximately 20% salt content) during and after pregnancy gained more weight and produced more wool than lambs from control ewes that grazed pasture during and after pregnancy, when offered saltbush later in life [46]. Lambs that experienced saltbush early in life showed lower rennin activity and higher excretion of salt from the body than inexperienced lambs [62].

Intake of sagebrush (*Artemisia tridentata*), a shrub that contains a high concentration of terpenes, is influenced by experience. Sheep that grazed sagebrush and understory vegetation at a high stocking density spent more time grazing on sagebrush during a conditioning phase, and thereafter showed a higher preference for sagebrush than sheep that grazed sagebrush and understory vegetation at a low stocking density during conditioning [63]. The proposed explanation is that when animals are encouraged to eat both palatable and unpalatable plants in an area, they are more likely to learn to eat mixtures of plants that mitigate toxicity. Similarly, cattle challenged to eat sagebrush supplemented with energy and protein—to enhance detoxification processes and better enable animals to eat sagebrush [64]—ate more sagebrush later in life and showed better performance than naïve cattle [65]. Another example on how nutrient–toxin interactions can attenuate the negative post-ingestive effects of toxic plants and enhance preference for chemically-defended plants comes from a recent study with wild rocket and sheep [23]. Wild rocket (*Diplotaxis tenuifolia*) is a nutritive plant, but it contains glucosinolates that cause intake depression in ruminants [66]. However, when sheep experienced wild rocket supplemented with protein, they showed an increased intake compared to the unsupplemented control animals. The total concentration of serum proteins and albumins were greater in sheep fed with protein supplements, which probably elicited a protective effect on biologically active by-products (isothiocyanates, thiocyanates, and nitriles, among others) derived from glucosinolate hydrolysis. Thereafter, when foraging behaviour was evaluated in an experimental arena where sheep could select from randomly distributed feeders containing either wild rocket or barley, the experienced sheep spent more time and ingested more wild rocket than the inexperienced sheep [23].

Exposure to toxins may modify the animal’s detoxification system or gut microbiome in ways that enhance toxin tolerance. For instance, prior exposure to plant toxins altered the diversity and population structure of the gut microbiome in woodrats (*Neotoma bryanti* and *N. lepida*), facilitating an increase in the abundance of genes that metabolize toxic compounds [67,68]. Moreover, herbivores increase the production enzymes in their tissues that detoxify plant toxins (i.e., cytochrome P450s) as a function of their previous exposure to these chemicals [69,70]. Thus, this information suggests that there is a potential optimal level of exposure to toxin-containing plants that modulates the intensity of the penalties associated with ingesting these unpalatable resources and, as a consequence, their preference by herbivores. In support of this, lambs exposed to food with oxalates, terpenes, and condensed tannins early in life consume greater amounts of these toxin-containing foods later in life than naïve animals, even when nutritious alternatives were available for consumption [71]. Chronic exposure to terpenes in sheep increases their ability to consume terpenes, as microbial populations in the rumen environment adapt to these toxins [72].

## 5. Experience and the Gut Microbiome

Emerging evidence suggests a significant crosstalk between the epigenome and the microbiome in human and animal models [73,74]. Gut microorganisms may promote epigenetic changes (e.g., DNA methylation, histone modifications, expression of non-coding RNAs) in intestinal epithelial cells with long-term consequences to the host. For instance, commensal bacteria contribute to intestinal homeostasis by maintaining their symbiosis with the intestinal immune system through epigenetic modification of the host gene [75]. In turn, diet can structure the host’s intestinal microbiome via changes in substrate availability, such as protein versus carbohydrates [76] or fat [77]. High-fat maternal or postnatal diets strongly influence the offspring’s intestinal microbiome in primates and human subjects, with implications for the maintenance of health later in life [77]. This dynamic interaction between the epigenome and gut microbes is starting to be revealed in ruminants, which represent a special case to explore such relationships given the pivotal influence of bacterial populations on the digestion of plant fibre. The rumen is the first gastrointestinal structure to be exposed to diet after ingestion, and thus the influence of diet on ruminal microbiome composition is even more pronounced than that observed in the colon microbiome of monogastric animals [78,79]. Changes in rumen microbial community structure promoted by diet are mediated by fluctuations in nutrient composition and in the redox potential of the rumen fluid [79]. In the mutualistic relationships between microorganisms and ruminants, natural selection can work to increase the fitness in both directions. Thus, the host may influence the microbiome through diet and feeding behaviour [80], and the microbiome in turn may influence the host through activation of epigenetic processes passed from generation to generation [81]. For instance, it has been proposed that the need to acquire a proper gut microbiome likely led to the evolution of social behaviour in herbivores, increasing the likelihood of bacterial exchange between mother and offspring and among members of the same social group [81]. Once established in the host, the microbiome may then interact with and alter the host epigenome, as previously described for human and animal models [73,74,75]. As an example, the development of a large rumen in response to high-fibre diets could be partially explained by the host-microbiome interaction, which increases the fitness of the microbes themselves and as a consequence the fitness of the host [81]. Collectively, the dynamic crosstalk between the epigenome and the microbiome is revealing that dietary experiences influencing the microbiome have the potential to structure the consumer’s phenotype with vertical transmission to subsequent generations. Low-quality diets may then promote changes in the microbiome, which in turn influence the physiology and behaviour of consumers and their offspring. New research focused on using animals as “landscape architects” to efficiently utilize unpalatable resources in the environment should focus on understanding how such diets influence gut community structure and how this change impacts phenotypes across generations.

## 6. Use of Toxin-Containing Feed to Combat Disease

Animals reduce internal parasites by selecting food high in primary and secondary compounds. Sheep with parasite burdens increase the intake of needed nutrients [82]. This process allows a herbivore to cope with the nutrient drain experienced during infection while enhancing the animal’s immune response [83,84]. Animals also increase their intake of secondary compounds for medicinal benefits [85]. Tannins are anti-diarrheal, antiseptic, anti-bacterial, anti-fungal, and anthelmintic [86,87,88]. Livestock fed plants with tannins (or dosed with tannins) have less nematodes and lower faecal egg counts than livestock without tannins and they gain more weight [83,89,90,91,92]. Tannins can enhance nutrition by increasing the supply of by-pass protein [93,94], which enhances immune responses to parasites [83]. Given the opportunity, parasitized sheep self-medicate with tannins to reduce helminthoses, even when the tannins are mixed in grape pomace that provides no nutritional benefits [95]. Sheep with high parasite burdens have an increased preference for a tannin-containing food compared with non-parasitized sheep until their parasite infection is terminated by dosing with ivermectin, a drug that kills internal parasites [96]. As parasite loads increase, sheep eat more tannin [97], and the faecal egg counts decrease as a result of this behaviour. If early exposure to secondary compounds enhances the ability of ruminants to cope with this challenge, while increasing the amounts that can be ingested voluntarily, then exposures to medicinal compounds may represent a novel alternative to enhance the bioactive dose that combats disease in ruminants. In addition, it has been found that the mother’s presence enhances the ability of lambs to self-medicate and that the process is bi-directional, since the presence of the offspring also enhances the ability of the mother to self-medicate [98]. These principles may be used to optimize the incorporation of medicinal compounds into ruminants’ diets when challenged by disease.

## 7. Negative Consequences of Being Exposed to an Unpalatable Feed

So far, we have dealt with positive experiences of unpalatable feed that increases the intake of and preference for them. However, the consequences of being exposed to unpalatable feed can be also negative, eliciting avoidance behaviour. For instance, the alimentary context in which animals experience a low-quality food can depress its subsequent use. Sheep exposed to low-quality feed (oat straw) early in life simultaneously with high levels of high-quality feed (sunflower meal and corn grain) ate less oat straw later in life than inexperienced sheep [99]. In a subsequent study with the same animals, it was demonstrated that experienced sheep had devalued oat straw as a consequence of the negative contrast that was developed through continuous comparison with the high level of the high-quality food experienced early in life [100].

When eating in excess, toxin-containing feed can induce strong and persistent aversions in ruminants, which result from stimulation of the emetic system of the midbrain and brain stem [101]. Within a 1-h meal, naïve goats learn to limit the intake of the current-season’s twigs of blackbrush that contain high levels of condensed tannin, causing a strong aversion [102,103]. The negative experience modifies the feeding behaviour of goats such that they prefer to eat older growth twigs of blackbrush, which although lower in nutrients are also lower in the condensed tannins that caused malaise [104,105]. In addition, sheep that experienced the toxic effects of glucosinolates in wild rocket ingested less than half the amount of the wild rocket consumed by the control animals when grazing in an experimental arena where they could select between the toxic feed and barley grain [23]. It was concluded that a negative feeding experience with wild rocket is needed for animals to display the typical pattern of aversion commonly observed in grazing situations. Toxin-containing feed can also cause tissue damage, adversely affecting animal health. For example, some saponins readily increase the permeability of the small intestinal mucosal cells, thereby facilitating the uptake of materials to which the gut would normally be impermeable [106,107]. Consistent with this notion, early exposure to terpene-containing sagebrush did not influence the use of sagebrush by lambs later in life [108]. When high-quality forages like alfalfa were highly available in the environment, lambs showed negligible values of sagebrush intake regardless of the previous level of sagebrush exposure. In fact, exposure to sagebrush by naïve lambs during testing had a stronger impact on sagebrush intake than in utero and after birth experiences [108]. The extent and duration of the exposure certainly had an impact on this outcome, as it is likely that exposure to terpenoids early in life negatively impacted the ability of lambs to cope with increasing concentrations of the toxin-containing shrub in their diets. These results are in contrast to those discussed above regarding early experience with sagebrush by cattle [65], where exposure enhanced heifers’ ability to consume sagebrush. This highlights the fact that information regarding herbivore exposure to plant toxins and their subsequent physiological and behavioural responses is limited [53]. More research is needed to develop a clear parameterization regarding the optimal dose and extent of exposure of herbivores to specific plant secondary compounds. Such studies would unveil the conditions under which exposure yields positive physiological and behavioural adaptations that allow animals to improve the efficiency of utilization—and consequently the efficiency of detoxification—of specific toxin-containing plants.

## 8. “Directions” of the Feeding Responses as a Function of Experience

In summary, amount, frequency, age, and context of experience while eating unpalatable food determine ruminant feeding behaviour towards those food types. Eating a low-quality or toxin-containing food too frequently or in excess may cause malaise and the development of food aversions with an expected decrease in preference for that food. In contrast, eating an unpalatable food in amounts or frequencies below the aversion threshold may condition a preference for the unpalatable resource.

Experience with unpalatable food early in life, when neurological/physiological and morphological processes are more amenable to change and adaptation to the environment, is expected to enhance preference for unpalatable resources. Similarly, the provision of nutrients to low-quality or toxin-containing food during the conditioning process may attenuate nutrient deficits or enhance detoxification, respectively, which is predicted to enhance the conditioning of preferences for unpalatable feed.

## 9. Evolutionary Consequences of Incorporating Unpalatable Plants in the Diet

From an optimality point of view, when palatable plants are freely available it is expected that animals will select them and reject unpalatable ones (optimal diet model [109]). However, a prediction from the optimal foraging theory is that herbivores are sensitive to the foraging cost of the preferred food [110,111]. Moreover, learning plays an important role in the foraging decision-making process [109], since the post-ingestive consequences from the ingesting unpalatable plants are improved when mixed in a diet with palatable plants [71].

Early experience can enhance the fitness value of a particular feed through an improved “extraction of nutrients” from unpalatable resources (e.g., due to an enhanced utilization of N or increased digestibility) [4]. In addition, foraging costs incurred during the acquisition of high-quality resources may be high under certain environmental conditions (i.e., increased searching and handling times due to low forage availability or increased effort to access high-quality resources). Collectively, this suggests that early experience with unpalatable food may represent a significant “tool” to enhance the fitness of consumers when availability of high-quality food is limited or when a combination of palatable and unpalatable resources leads to synergistic associations that increase nutrient gain per unit of effort. Some evidence in this regard exists for low-quality feed [60,61] or for toxin-containing plants [63] when palatable plants become less accessible.

## 10. Plant Community Consequences of Incorporating Unpalatable Plants in the Diet

Unpalatable plant encroachment is a widespread phenomenon in diverse ecosystems all over the world [112]. Selective grazing of palatable plants increases the competitive advantages of unpalatable plants, favouring their establishment and growth [113,114,115,116,117,118]. Animals that learn the benefits of incorporating plants differing in palatability in their diet may achieve more uniform use of different species at plant patch or plant community level, counteracting the process of species replacement and depletion of floristic diversity.

If animals start including unpalatable plants in the diet when palatable plants are too scarce, they are unlikely to learn the potential benefits of mixing alternative food. Therefore, a more uniform use of plant species is not realized. However, if herbivores are encouraged to mix food in a way to overcome the nutritional and/or toxic limitations of unpalatable food, they are more likely to acquire diet mixing behaviour and develop an increased preference for, and intake of, unpalatable food [23,60,61,64,71,119,120]. Cattle supplemented with energy and protein learned to eat sagebrush and showed increased use of the shrub compared to naïve cattle when grazed on a sagebrush steppe; the grazing behaviour of experienced cattle decreased the abundance of sagebrush and increased the abundance of grasses and forbs [65].

## 11. Behaviour-Based Management

The possibility of altering behaviour through experience to better adapt animals to forage in the habitat in which they are reared has been scarcely considered in planning the use of pastures and rangelands. However, as illustrated above, basic knowledge on behavioural processes can be translated into behavioural applications that provide low-cost solutions to many challenges that producers face in managing sustainable livestock production systems (Figure 1). For instance, exposure in utero and early in life with their mothers to food that animals will encounter later in life can be used to improve the performance of replacement females, stockers, and finishers under free or confinement conditions. Exposure early in life with their mothers can also be used to change habitat preference and minimize damage of fragile and/or highly preferred habitats (e.g., riparian areas). On the other hand, the facilitation of learning the associations between an unpalatable plant flavour and improved post-ingestive consequences from nutrient supplementation, or mixing with palatable plants under high stock density grazing, can be used to increase the utilization of unpalatable plants and improve ecosystem biodiversity and animal functioning.

## 12. Conclusions

Experimental evidence supports the argument that environmental experiences help ruminants to better cope with unpalatable forages. Experience is especially critical early in life, when body functions are more amenable to change. Experience alters morpho-physiological mechanisms toward improving the post-ingestive consequences of both nutrient-deficient food and toxin-containing food. Furthermore, experiential learning provides flexibility in diet selection, which is critical in changing foraging environments. The practical challenge is to develop and apply behavioural solutions to problems faced by animal production and the integrity of the lands in systems that include unpalatable forage resources. By understanding and applying behavioural principles, it is possible to better adapt livestock that is going to thrive on the use of unpalatable forages throughout their lives. In addition, a more uniform use of resources can be achieved from the landscape level down to the individual plant, with the consequent benefits to ecosystem integrity and stability.
